# SP1–DLEU1–miR-4429 feedback loop promotes cell proliferative and anti-apoptotic abilities in human glioblastoma

**DOI:** 10.1042/BSR20190994

**Published:** 2019-12-06

**Authors:** Xiaolei Liu, Ruwei Chen, Lijun Liu

**Affiliations:** 1 Department of Neurosurgery, Xianyang Hospital of Yan’an University, Xianyang City, Shaanxi Province 712000, P.R. China; 2 Department of Neurosurgery, Binzhou People’s Hospital, Shandong Province 256610, P.R. China; 3 Department of Neurosurgery, Xiangyang No. 1 People’s Hospital Affiliated to Hubei University of Medicine, Xiangyang 441000, Hubei, P.R. China

**Keywords:** DLEU1, feedback loop, glioblastoma, miR-4429, SP1

## Abstract

Mounting studies have revealed that long non-coding RNA (lncRNA) deleted in lymphocytic leukemia 1 (DLEU1) positively regulated the initiation and development of various human malignant tumors. Nevertheless, the function and mechanism of DLEU1 in human glioblastoma multiforme (GBM) remain elusive and ill-defined. The current study was designed to highlight the functional role and disclose the underlying molecular mechanism by which DLEU1 regulated GBM development. We found that DLEU1 was up-regulated in GBM and DLEU1 knockdown significantly inhibited GBM cell proliferation and induced apoptosis. As predicted by bioinformatics analysis and validated in mechanistic assays, SP1 could bind to the promoter region of DLEU1 to activate DLEU1 transcription. Additionally, miR-4429 was verified as a target gene of DLEU1 and negatively modulated by DLEU1. More importantly, miR-4429 overexpression repressed the mRNA and protein levels of SP1 via binding to the 3′UTR of SP1. Overexpression of SP1 or miR-4429 inhibitor could partly abolish the effect of DLEU1 knockdown on cell viability and apoptosis in GBM. Accordingly, our experimental data revealed that SP1–DLEU1–miR-4429 formed a feedback loop to promote GBM development, providing a new evidence for the role of DLEU1 in GBM.

## Introduction

Tumor derived from neuroepithelium is collectively referred to as glioma. As a common intracranial malignancy, glioma is classified into four groups. Based on cell morphology, it includes astrocytoma, oligodendroglioma, ependymoma and mixed glioma [1]. Statistically, glioblastoma multiforme (GBM) accounts for at least 80% of all astrocytoma cases [2]. The high proliferative rate leads to the fate that GBM patients only survive for approximately 15 months after diagnosis [3–5]. Currently, GBM patients benefit from personalized therapy for molecular targets, but mostly results are unsatisfactory or strong side effects are caused. Hence, exploring novel biomarkers is urgently needed for GBM treatments.

Non-coding RNAs (ncRNAs) include long ncRNA (lncRNA), microRNA (miRNA) and circular RNA (circRNA), acting essential regulatory roles in human diseases, particularly in tumors [6,7]. Among them, lncRNA is defined as the highly conserved RNA transcripts without protein-coding potential [8]. LncRNA consists of more than 200 nucleotides (nt) [9]. Based on existing studies, lncRNA is widely identified to modulate diverse biological behaviors and cellular processes in GBM, thereby functioning as a crucial regulator in the GBM development [10–12]. LncRNA deleted in lymphocytic leukemia 1 (DLEU1) was confirmed to be up-regulated in colorectal cancer [13], oral squamous cell carcinoma [14], ovarian cancer [15], pancreatic ductal adenocarcinoma [16] and gastric cancer [17], consistently exerting oncogenic role. Recently, transcription factor is reported to activate lncRNA transcription, thereby promoting lncRNA expression in tumors [18–20]. Therefore, our study investigated the bio-function and upstream mechanism of DLEU1 in GBM cells.

As an indispensable member in ncRNAs family, the functional and mechanistic involvements of miRNA are extensively explored in numerous tumors [21–23]. It is well-known that miRNA regulates gene expression by binding to the 3′-untranslated region of target mRNA [24]. Mounting reports disclose that lncRNA functions as a competing endogenous RNA (ceRNA) or a molecular sponge to modulate expression and bio-function of miRNA, suppressing miRNA modulation on mRNA [25–27]. However, it remains elusive whether DLEU1 affects GBM progression in ceRNA manner.

In the present study, we confirmed that DLEU1 was up-regulated in GBM and DLEU1 knockdown notably repressed GBM cell viability and induced cell apoptosis. Transcription factor SP1 was identified as an upstream activator of DLEU1 and a downstream target gene of miR-4429. More importantly, DLEU1 could regulate SP1 expression via sponging to miR-4429, forming a feedback loop. Collectively, our experimental results provide a potential therapy target for patients with GBM.

## Materials and methods

### Bioinformatics analysis

The Cancer Genome Atlas database (TCGA; http://gepia.cancer-pku.cn/index.html) was applied to acquire the expression profiles of DLEU1 and SP1 in glioblastoma. The transcription factors of DLEU1 were predicted in University of California Santa Cruz (UCSC Genome Browser; http://genome.ucsc.edu/). The motif and potential binding sites of SP1 within the DLEU1 promoter region were obtained from JASPAR tool (http://jaspar.genereg.net/). Starbase (http://starbase.sysu.edu.cn/) was utilized to search out the putative miRNAs targeted by DLEU1. The binding sites of miR-4429 and SP1 were predicted by TargetScan (http://www.targetscan.org/vert_72/).

### Cell lines and culture

U251, U87 and LN229, the three most acknowledged human glioblastoma cell lines along with a normal brain glial cell line HEB were commercially obtained from American Type Culture Collection (ATCC, Manassas, VA, U.S.A.). The four cell lines were cultured in Dulbecco’s Modified Eagle’s Medium (DMEM, Gibco, San Francisco, CA, U.S.A.) at 37°C. Culture medium was placed in a humid incubator with 5% CO_2_. Ten percent fetal bovine serum (FBS, Gibco) and 1% penicillin–streptomycin (HyClone, Logan, UT, U.S.A.) were utilized to supplement medium. Cell passage was carried out while cells grew up to 80–90% confluence. The replacement of culture medium was repeated every 3 days.

### RNA isolation and quantitative real-time polymerase chain reaction

Isolation of total RNA from cultured cells was made by using TRIzol® Reagent (Thermo Fisher Scientific, Waltham, MA, U.S.A.) as per the guidelines provided by the supplier. Cells were washed with phosphate-buffered saline (PBS, Bio-Rad Laboratories, Inc., Hercules, CA, U.S.A.) after transfection for 48 h. Total RNA was denatured at 65°C for 5 min and then reverse transcribed into complementary DNA (cDNA) using Reverse Transcription Kit (Toyobo, Osaka, Japan). The cDNA templates were amplified via real-time RT-PCR using the SYBR Green PCR Master Mix (TaKaRa, Tokyo, Japan). The thermal cycling conditions were described as follows: 95°C for 5 min, 40 cycles of 95°C for 30 s, 60°C for 30 s and 72°C for 1 min, following the final extension at 72°C for 5 min. The following specific primers were used in the present study: DLEU1 forward primer 5′-TGCATTTAAAACCGCCCTGC-3′, reverse primer 5′-TTGAAGAAGGAGACCACGCC-3′; SP1 forward primer 5′-GCAAGCTTTCCATGCACCC-3′, reverse primer 5′-AAGGCGAAAGCCACCAGTAT-3′; miR-4429 forward primer 5′-AGAGAGAAAAGCAGGGCTGAGA-3′, reverse primer 5′-CTCTACAGCTATATTGCCAGCCA-3′; GAPDH forward primer 5′-GGAAAAGGACATTTCCACCGC-3′, reverse primer 5′-TCCCGGTGACATTTACAGCC-3′; U6 forward primer 5′-ACGACAAACCTGCTGGTAGC-3′, reverse primer 5′-TCTGGACGAAGAGGATTCGC-3′. GAPDH and U6 were seen as the internal reference. The relative expression of RNA was calculated via the comparative 2^−ΔΔ*C*_t_^ method.

### Cell transfection

U251 and LN229 cell lines were planted into six-well plates with DMEM containing 10% FBS and incubated at 37°C. After culturing cells to 80% attachment rate, cell transfection was performed with Lipofectamine 2000 (Invitrogen, Carlsbad, CA, U.S.A.) following the user guide. To silence DLEU1 expression, cells were transfected with the short hairpin RNAs specific to DLEU1 (sh-DLEU1#1, sh-DLEU1#2 and sh-DLEU1#3, GeneCopoeia, Guangzhou, China). Non-specific shRNAs (GeneCopoeia) were seen as the negative control (sh-NC). The pcDNA3.1 vectors targeting DLEU1 and SP1 (pcDNA-DLEU1 and pcDNA-SP1) were designed and synthesized by Shanghai GenePharma Co. Ltd. (Shanghai, China). The empty pcDNA3.1 vectors were used as the control (NC). The miR-4429 mimics (RiboBio, Guangzhou, China) or inhibitors (GenePharma) was transfected into U251 and LN229 cells to enhance or knockdown miR-4429 expression. Forty-eight hours later, the transfected cells were reaped and used for subsequent experiments.

### Cell counting kit-8 assay

The transfected U251 and LN229 cells were harvested and diluted at a density of 1 × 10^3^ cells/ml prior to planting into 96-well plates. The serum-free medium was replaced every third day. Ten microliters of CCK-8 solution (Dojindo Molecular Technologies, Inc., Kumamoto, Japan) were added and incubated with cells for 1 h at 37°C with 5% CO_2_. After cells were cultured for 24, 48, 72 and 96 h, OD value at 450 nm was assessed using microplate reader (Bio-Rad, Hercules, CA, U.S.A.). Experimental results were obtained from three independent replicates.

### EdU incorporation assay

After transfection, U251 and LN229 cells were seeded at 500 cells/well in 96-well plates and cultured at 37°C with 5% CO_2_ for 2 days. One hundred microliters of 50 μM 5-ethynyl-20-deoxyuridine (EdU) medium diluent was added and incubated with cells for 3 h. Thereafter, cells were fixed in 4% paraformaldehyde before treatment with 0.5% Troxin X-100 for 10 min. One hundred microliters of 1× Apollo® 488 fluorescent staining reaction liquid was mixed with cells for half an hour at 37°C with 5% CO_2_. Cell nuclei were labeled by DAPI (Sigma–Aldrich, St. Louis, Missouri, U.S.A.) in the dark. Experimental data were expressed as the percentage of EdU-positive cells (green) relative to total DAPI-positive cells (blue). EdU assay was repeated independently more than three times.

### Caspase-3 activity detection

The Caspase-3 Assay Kit (Colorimetric, Abcam, Cambridge, MA, U.S.A.) was utilized to evaluate caspase-3 activity in accordance with the user manual. The transfected cells (5 × 10^6^) were cultured in six-well plates and suspended in 50 μl of pre-chilled Cell Lysis Buffer, followed by isolating cell supernatant and assessing protein concentration. Afterward, 50 μl of 2× Reaction Buffer and 5 μl of 4 mM DEVD-p-NA substrate (at 200 μM final concentration) were incubated with cells for 2 h. Finally, the absorbance at the wavelength of 405 nm was measured by a microplate reader (Tecan, Männedorf, Switzerland). Caspase-3 activity was detected thrice in our study.

### Western blot assay

Total proteins were isolated from cells using RIPA buffer reagent (Thermo Fisher Scientific, U.S.A.) and separated using sodium dodecyl sulfate/polyacrylamide gel electrophoresis (SDS/PAGE). After transferring on to polyvinylidene fluoride (PVDF) membranes, proteins were blocked in 5% bovine serum albumin (BSA) for 2 h at room temperature. Thereafter, the membranes were incubated with specific primary antibodies (1:1000 dilution) overnight at 4°C. All primary antibodies, including anti-Bax (ab32503), anti-Bcl-2 (ab32124), anti-SP1 (ab227383) and anti-GAPDH (ab9483) were purchased from Abcam (Cambridge, MA, U.S.A.). After thrice washing with Tris-buffered saline with Tween (TBST), the PVDF membranes were incubated with secondary antibodies conjugated to horseradish peroxidase (1:2000 dilution, Abcam) for 2 h in the dark. The membranes were visualized via an enhanced chemiluminescence (ECL) detection system (Bio-Rad lab, Hercules, CA, U.S.A.). GAPDH was taken as an internal reference. Western blot assay was independently conducted in triplicate.

### Chromatin immunoprecipitation assay

The chromatin immunoprecipitation (ChIP) assay was carried out in U251 and LN229 cell lines by the use of the Magnetic ChIP Kit (Pierce, Rochford, IL, U.S.A.). Cells (5 × 10^6^) were incubated in Matrigel and cross-linked with 1% formaldehyde. After lysing in the lysis buffer, the nuclear chromatin was isolated and subjected to sonication. The chromatin diluent was cultured with Protein-A/G beads. Thereafter, the complexes (DNA–protein) were immunoprecipitated with antibodies against SP1 and immunoglobulin G (IgG), followed by treatment with beads. After eluting and purifying, the precipitated DNA was analyzed by quantitative real-time polymerase chain reaction (qRT-PCR). ChIP assay result was acquired from three different replications.

### Luciferase reporter assay

Dual-Luciferase Reporter Assay System (Promega Corp., Madison, WI, U.S.A.) was applied to conduct luciferase reporter assay. For DLEU1 promoter luciferase activity assay, the wild-type or mutant sequences of binding sites 3 of SP1 within DLEU1 promoter region (SITE 3-WT, SITE 3-MUT) were separately constructed by GenePharma (Shanghai, China). U251 and LN229 cell lines were seeded into 24-well plates and co-transfected with SITE 3-WT/MUT reporter plasmid and pcDNA-SP1 vector using Endofectin™-Plus (GeneCopoeia, Maryland, U.S.A.). Cells treated with the empty pcDNA3.1 vector were taken as the control (NC). To detect the interaction among DLEU1, miR-4429 and SP1, cells were transfected with DLEU1-WT/MUT or SP1-WT/MUT (Genepharma) along with miR-4429 mimics or miR-NC using Lipofectamine 2000 (Invitrogen). The relative luciferase activity was assessed by a luciferase assay kit (Promega Corp.), normalizing to that of *Renilla* activity. Three independent assays were performed.

### Subcellular fractionation assay

A total of 1 × 10^7^ U251 and LN229 cells were thrice rinsed in PBS and suspended in 500 μl of cytoplasmic extract buffer on ice for 20 min. NP40 containing 10 mM HEPES, 60 mM KCl, 1 mM EDTA, 0.075% (v/v) NP40, 1 mM DTT and 1 mM PMSF, pH 7.6 was added into the buffer. Following centrifugation at 1500×***g*** for 4 min, the cytosol fraction in supernatant was transferred into a clean tube. The remaining nuclear fraction was washed for three times and subjected to 100 μl of nuclear extract buffer containing 20 mM Tris/HCl, 420 mM NaCl, 1.5 mM MgCl_2_, 0.2 mM EDTA, 1 mM PMSF and 25% (v/v) glycerol, pH 8.0. After culturing on ice for 20 min and centrifuging at 12000×***g*** for 10 min, the insoluble fraction was removed. RNA was extracted from cytoplasmic or nuclear fractions using TRIzol® Reagent (Thermo Fisher Scientific) and examined by qRT-PCR. The expression of GAPDH and U6 were detected as cytoplasm and nucleus controls. Three replications of subcellular assay were conducted.

### RNA-immunoprecipitation assay

The Magna RIP™ RNA-Binding Protein Immunoprecipitation Kit (Millipore, Billerica, MA, U.S.A.) was applied to conduct RNA immunoprecipitation (RIP) assay. U251 and LN229 cells (1 × 10^7^) were reaped and rinsed in ice-cold PBS. Thereafter, cells were lysed in the RIP lysis buffer at 4°C for 30 min. Cell lysates (100 μl) were cultured with magnetic beads conjugated with human antibodies against argonaute 2 (Ago2) (Millipore, U.S.A.) or normal IgG (Millipore, U.S.A.). Proteinase K buffer was applied to digest proteins. The immunoprecipitated RNA and total RNA were extracted from the whole cell lysates (input control) and analyzed by qRT-PCR examination. Each experimental procedure was repeated more than three times.

### RNA-pull down assay

RNA-pull down assay was performed using a Pierce Magnetic RNA-Protein Pull-Down Kit (Thermo Fisher Scientific, Waltham, MA, U.S.A.) according to the standard method. Protein extracts from U251 and LN229 cells were cultured with biotin-labeled miR-4429 (Bio-miR-4429-wt and Bio-miR-4429-mut) and negative control (Bio-NC). Afterward, proteins were incubated with streptavidin agarose magnetic beads (Invitrogen) for 1 h at room temperature. The complexes bound to the beads were eluted with Biotin Elution Buffer and boiled in SDS buffer for 10 min. The relative fold enrichment of SP1 was evaluated by qRT-PCR analysis. All procedures of pull-down assay were carried out thrice.

### Statistical analysis

All statistical analyses were performed using the SPSS® vision 22.0 software package (IBM, Chicago, U.S.A.) and GraphPad Prism 5 (GraphPad Software, La Jolla, California, U.S.A.). Data were reported as means ± standard derivation (SD). Differences were assessed using Student’s *t* test (between two groups) and one-way analysis of variance along with post-hoc Tukey’s tests (for more than two groups). Two-sided *P*-value threshold was set as 0.05 to be statistically significant.

## Results

### DLEU1 knockdown inhibited GBM cell proliferation and induced apoptosis

**Figure 1:**
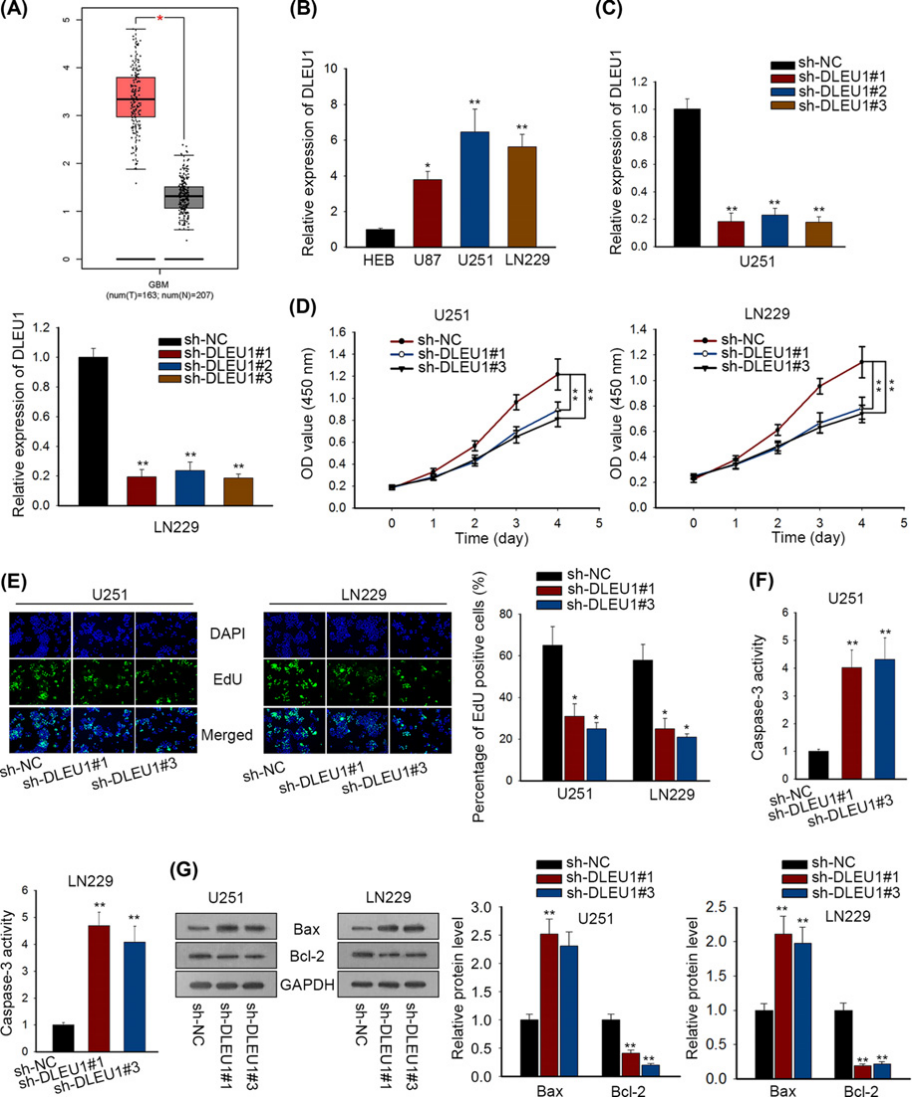
DLEU1 knockdown inhibited GBM cell proliferation and induced apoptosis (**A**) The expression pattern of DLEU1 in GBM was obtained from TCGA and indicated the up-regulation of DLEUL in GBM tissues relative to normal samples. (**B**) The expression levels of DLEU1 in GBM cells and a normal brain glial cell line HEB were examined by qRT-PCR and highly expressed DLEU1 was observed in GBM cells. (**C**) DLEU1 expression was silenced in U251 and LN229 cell lines through the transfections of shRNAs targeting DLEU1. (**D**) CCK-8 assay was used to detect GBM cell viability in response to DLEU1 depletion and disclosed a viability inhibition caused by DLEU1 depletion. (**E**) EdU assay was applied to assess the alteration of GBM cell proliferation after knocking down DLEU1 expression and found the proliferation suppression induced by DLEU1 knockdown. (**F**) GBM cell apoptosis was evaluated by caspase-3 activity detection; consequently, a promoted caspase-3 activity was disclosed. (**G**) Pro-apoptotic protein Bax was notably increased and anti-apoptotic protein was decreased responding to DLEU1 knockdown from Western blot assay. *P*<0.05* and *P*<0.01** are considered to be significant statistically.

TCGA database was applied to assess the expression pattern of DLEU1 in GBM. As illustrated in Figure 1A, DLEU1 was significantly up-regulated in GBM tissues (*n*=163) compared with the normal tissues (*n*=207). High expression level of DLEU1 was also observed in the three most acknowledged human glioblastoma cell lines U87 (*P*<0.05), U251 (*P*<0.01) and LN229 (*P*<0.01) in comparison with the normal brain glial cell line HEB, particularly in U251 and LN229 cells (Figure 1B). Consequently, we silenced DLEU1 expression in U251 and LN229 cells to evaluate the bio-function of DLEU1 in GBM. DLEU1 expression was successfully depleted by specific shRNAs in U251 (*P*<0.01) and LN229 cells (*P*<0.01), especially by sh-DLEU1#1 and sh-DLEU1#3 (Figure 1C). Therefore, sh-DLEU1#1 and sh-DLEU1#3 were utilized to conduct subsequent functional assays. CCK-8 assay revealed that cell viability in U251 (*P*<0.01) and LN229 cells (*P*<0.01) was obviously inhibited following DLEU1 depletion (Figure 1D). The number of EdU-positive cells showed that DLEU1 knockdown repressed cell proliferative ability of U251 (*P*<0.05) and LN229 cells (*P*<0.05) (Figure 1E). As validated previously, activation of caspase-3 predicted cell apoptosis promotion. Therefore, we conducted caspase-3 activity assay to examine GBM cell apoptosis following DLEU1 depletion. As shown in Figure 1F, caspase-3 activity was enhanced via the transfection with sh-DLEU1#1 and sh-DLEU1#3 plasmids in U251 (*P*<0.01) and LN229 cells (*P*<0.01), indicating that declined DLEU1 expression induced more GBM cell apoptosis. It was well elucidated that the apoptotic process of cells was under the modulation of Bcl-2/Bax family protein [28,29]. Therefore, we assessed the apoptosis-related protein levels (Bcl-2, Bax) by Western blotting. We observed that the level of pro-apoptotic protein Bax was notably increased responding to DLEU1 knockdown in U251 and LN229 cells, whereas the level of anti-apoptotic protein Bcl-2 was distinctly decreased after silencing DLEU1 expression in in U251 and LN229 cells (Figure 1G). Overall, our findings pointed that DLEU1 as a positive regulator of GBM cell growth and negative regulator of cell apoptosis.

### The transcription factor SP1 contributed to the up-regulation of DLEU1 in GBM

**Figure 2:**
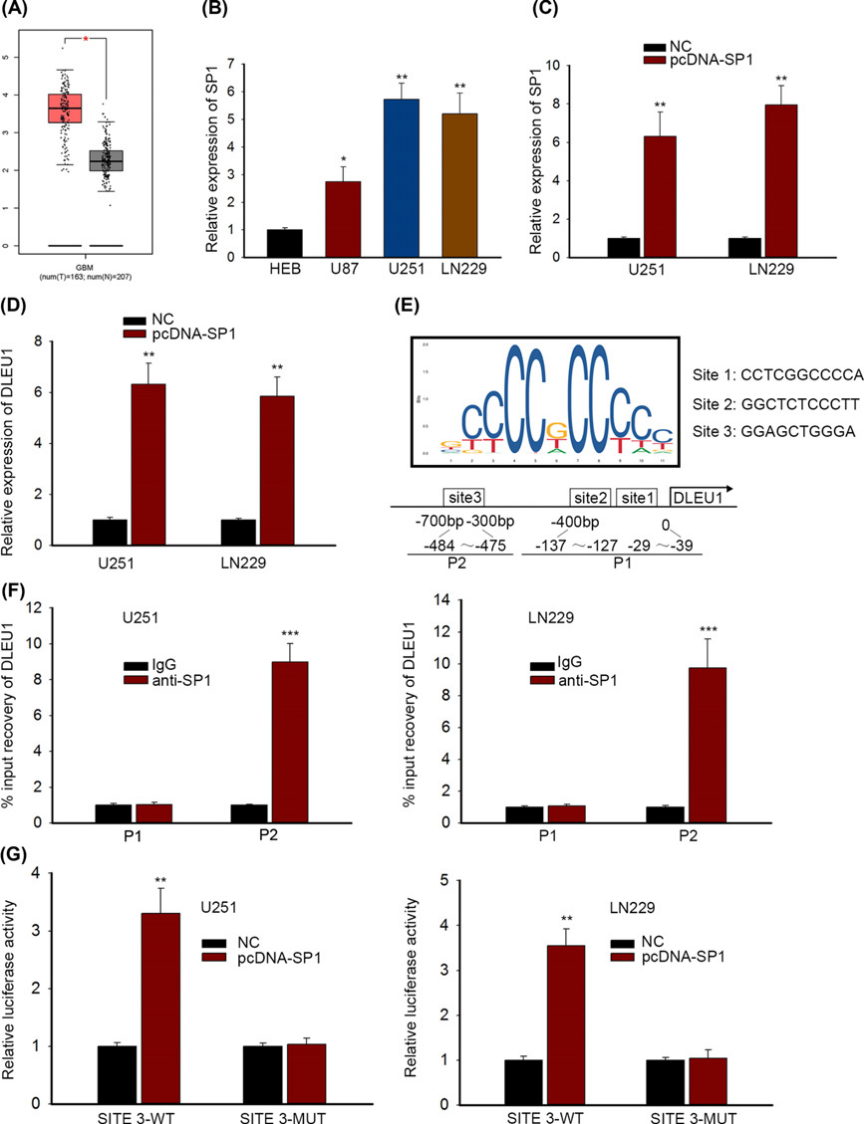
The transcription factor SP1 contributed to the up-regulation of DLEU1 in GBM (**A**) The expression profile of SP1 in GBM was acquired from TCGA dataset and suggested an elevation of SP1 in GBM tissues. (**B**) SP1 was highly expressed in GBM cells comparing with that in HEB cell from qRT-PCR analysis. (**C**) SP1 expression was overexpressed in U251 and LN229 cells after the transfection of SP1 overexpressing plasmids in qRT-PCR assay. (**D**) Overexpression of SP1 enhanced DLEU1 expression in GBM cells in qRT-PCR assay. (**E**) The motif and putative binding sites of SP1 in DLEU1 promoter region were found in JASPAR. (**F**) ChIP assay was used to probe whether fragment P1 or P2 was responsible for binding to DLEU1 promoter region and P2 was validated as the actual site. (**G**) Luciferase reporter assay revealed the impact of SP1 overexpression on the wild-type or mutant form of putative binding sites 3 of DLEU1 promoter and SITE3-WT was enhanced by upon pcDNA-SP1 transfection. *P*<0.05*, *P*<0.01** and *P*<0.001*** were considered to be significant statistically.

Although we found the high expression of DLEU1 in GBM, the regulatory mechanism involved in DLEU1 up-regulation remained elusive. Existing evidences report that several key transcription factors contribute to the up-regulation of lncRNA in human cancers, such as SP1, ELK1, STAT3 and YY1 [30–34]. Using UCSC tool, SP1 was predicted as a transcription factor of DLEU1. Importantly, the expression level of SP1 in 163 GBM tissues was higher than that in 207 normal tissues from TCGA (Figure 2A). Similar to DLEU1 expression, SP1 expression was up-regulated in GBM cell lines (*P*<0.05, *P*<0.01; Figure 2B). Hence, we speculated that SP1 could positively regulate DLEU1 through inducing its transcription. Furthermore, we detected the effects of SP1 overexpression on DLEU1 level. Overexpressed SP1 expression was observed through transfecting pcDNA-SP1 into U251 and LN229 cells (*P*<0.01; Figure 2C). qRT-PCR analysis showed that SP1 overexpression promoted DLEU1 expression in U251 and LN229 cells (*P*<0.01; Figure 2D). Hence, the expression level of DLEU1 was positively related to SP1 expression. Mechanically, as illustrated in Figure 2E, only three binding sites of SP1 in DLEU1 promoter region were predicted by JASPAR. The putative binding sites 1 (−29 to −39 bp: CCTCGGCCCCA) and sites 2 (−137 to −127 bp: GGCTCTCCCTT) were included in fragment P1 (0 to −400 bp). The fragment P2 (−300 to −700 bp) contained the potential binding sites 3 (−484 to −475 bp: GGAGCTGGGA). ChIP assay demonstrated that the fragment P2 was responsible for the binding of SP1 to the DLEU1 promoter region in U251 and LN229 cells (*P*<0.001; Figure 2F). Luciferase reporter assay suggested that the relative luciferase activity of binding sites 3 wild-type (SITE 3-WT) was enhanced upon pcDNA-SP1 transfection in U251 and LN229 cells (*P*<0.01), but when the binding sites 3 was mutated, the promotion of luciferase activity disappeared (SITE 3-MUT) (Figure 2G). Taken together, DLEU1 was transcriptionally up-regulated by SP1.

### DLEU1 bound to miR-4429 in GBM

**Figure 3:**
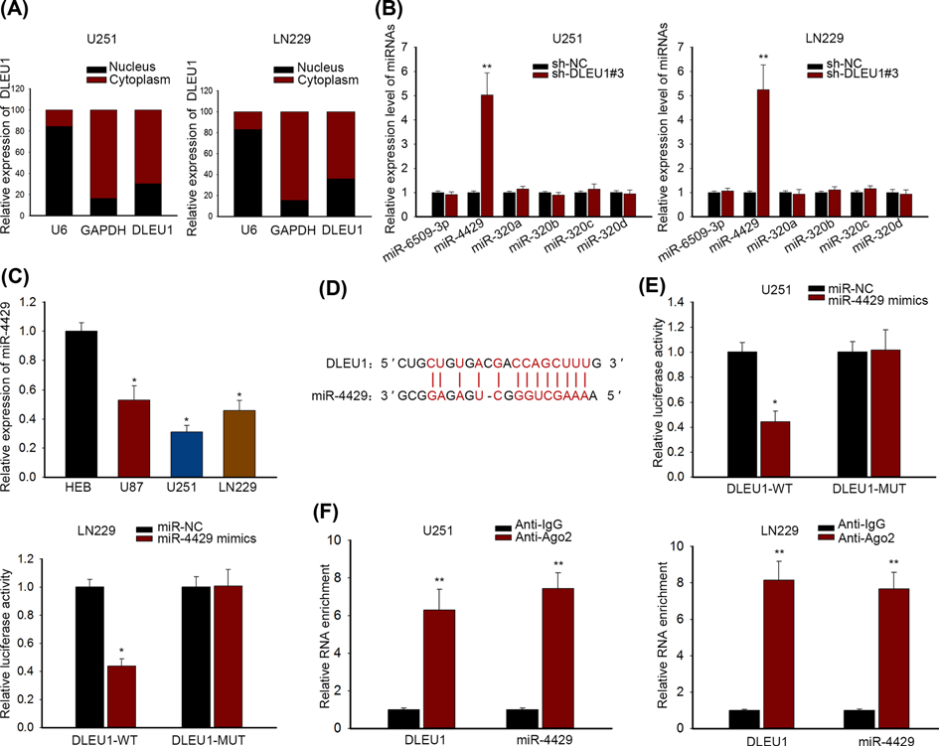
DLEU1 bound to miR-4429 in GBM (**A**) Subcellular fractionation assay proved that DLEU1 expression was more abundant in cytoplasmic fraction. (**B**) The distinct expression levels of six putative miRNAs were analyzed by qRT-PCR and miR-4429 was up-regulated upon DLEU1 silencing. (**C**) miR-4429 in GBM cells was lower than that in HEB cell in qRT-PCR assay. (**D**) The binding sites between DLEU1 and miR-4429 were predicted in Starbase tool. (**E**) The interaction of DLEU1 and miR-4429 was validated by luciferase reporter assay. (**F**) RIP assay with antibodies against Ago2 and IgG was carried out and proved the interaction of DLEU1 and miR-4429. *P*<0.05* and *P*<0.01** were considered to be significant statistically.

Influenced by the ceRNA notion, we further explored whether the DLEU1-mediated ceRNA mechanism was involved in the GBM development. Owing to the fact that cytoplasmic lncRNA could function as ceRNA, subcellular fractionation assay was implemented to unveil DLEU1 localization. It indicated that DLEU1 expression was more abundant in cytoplasmic fraction than that in nuclear fraction of GBM cells (Figure 3A), supporting that DLEU1 could affect gene expression post-transcriptionally. Afterward, we found six putative miRNAs (miR-6509-3p, miR-4429, miR-320a, miR-320b, miR-320c and miR-320d) which could be targeted by DLEU1 in Starbase dataset. qRT-PCR suggested that only miR-4429 expression was obviously strengthened following DLEU1 depletion in U251 cells (*P*<0.01; Figure 3B). Additionally, it was presented that miR-4429 was lowly expressed in GBM cells (*P*<0.05) (Figure 3C). Based on the predicted binding sites between DLEU1 and miR-4429 acquired from Starbase (Figure 3D), luciferase reporter assay was performed and disclosed that miR-4429 mimics significantly reduced the luciferase activity of DLEU1-WT in U251 and LN229 (*P*<0.05) cells, rather than that of DLEU1-MUT (Figure 3E). To confirm that DLEU1 and miR-4429 were associated with the RNA-induced silencing complex (RISC), of which the key component was Ago2, therefore, RIP assay was carried out using specific antibodies against Ago2 and IgG proteins. Result from RIP assay exhibited that Ago2 complex were enriched with DLEU1 and miR-4429 in in U251 and LN229 cells (*P*<0.01; Figure 3F). Collectively, DLEU1 interacted with miR-4429 by acting as its sponge.

### SP1 was a target gene of miR-4429 in GBM

**Figure 4:**
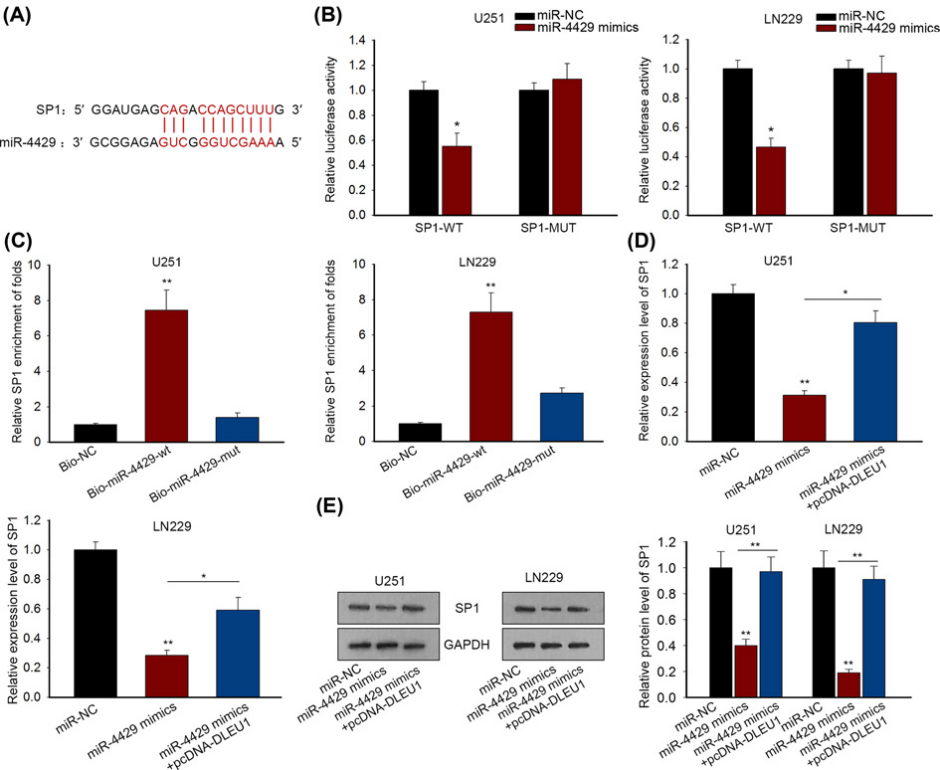
SP1 was a target gene of miR-4429 in GBM (**A**) The binding sites of miR-4429 and SP1 were predicted by TargetScan tool. (**B**) The interaction between miR-4429 and SP1 was verified by luciferase reporter assay. (**C**) Biotinylated RNA probes (Bio-NC, Bio-miR-4429-wt and Bio-miR-4429-mut) were applied to conduct RNA pull-down assay and validate the interaction of SP1 and miR-4429. (**D,E**) The mRNA and protein levels of SP1 were decreased after transfection with miR-4439 mimics while transfecting pcDNA-DLEU1 promoted SP1 mRNA and protein. *P*<0.05* and *P*<0.01** were considered to be significant statistically.

Considering that SP1 was up-regulated and related positively to DLEU1 expression in GBM, we intended to probe whether SP1 acted as a target gene of miR-4429, forming a DLEU1/miR-4429/SP1 ceRNA mechanism. As expected, we found the potential binding sites of miR-4429 in SP1 3′UTR using TargetScan (Figure 4A). Luciferase reporter assay revealed that luciferase activity of SP1-WT was weakened in response to miR-4429 mimics, no distinct alteration was found in luciferase reporter of SP1-MUT in U251 and LN229 cells (*P*<0.05; Figure 4B). Using biotinylated miR-4429 probes in RNA pull-down assay, we observed that SP1 mRNA was greatly pulled down by bio-miR-4429-wt in U251 and LN229 cells (*P*<0.01), further confirming the interaction between miR-4429 and SP1 (Figure 4C). More importantly, the mRNA level and protein level of SP1 were decreased by miR-4429 mimics, but restored by pcDNA-DLEU1 in U251 and LN229 cells (*P*<0.05, *P*<0.01; Figure 4D,E). These data indicated that DLEU1 sponged miR-4429 to promote SP1 expression.

### SP1–DLEU1–miR-4429 axis regulated cell proliferation and apoptosis in GBM

**Figure 5:**
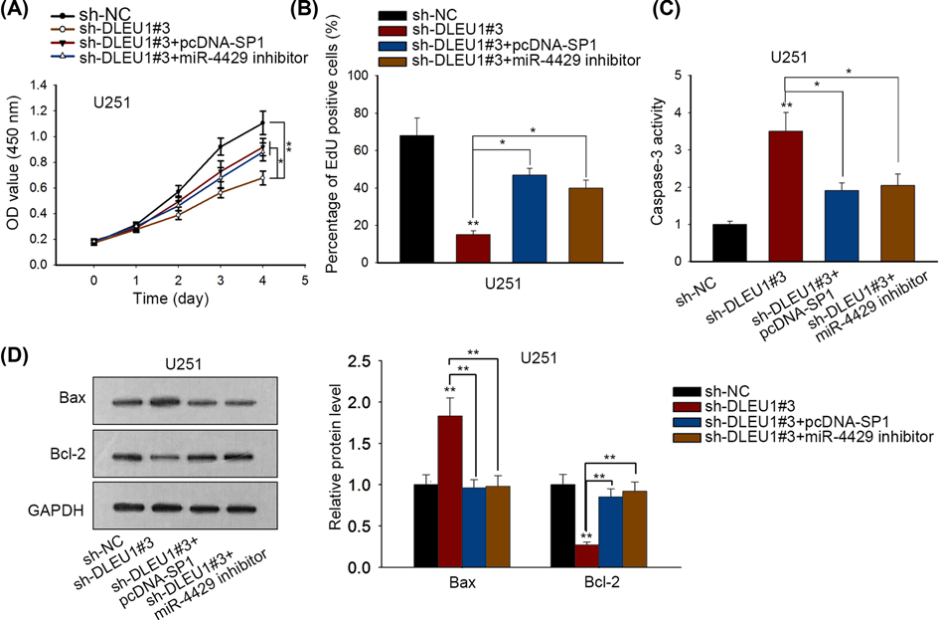
SP1–DLEU1–miR-4429 axis regulated cell proliferation and apoptosis in GBM (**A,B**) CCK-8 and EdU assays observed that pcDNA-SP1 or miR-4429 inhibitor rescued cell proliferation caused by sh-DLEU1#3. (**C**) Caspase-3 activity was enhanced by DLEU1 depletion, but abolished by SP1 overexpression or miR-4429 knockdown. (**D**) sh-DLEU1#3-induced Bax and Bcl-2 protein alteration were rescued by pcDNA-SP1 or miR-4429 inhibitor. *P*<0.05* and *P*<0.01** were considered to be significant statistically.

We designed and carried out rescue assays in U251 cell line to evaluate the impact of SP1–DLEU1–miR-4429 axis on GBM cell proliferation and apoptosis. CCK-8 assay suggested that GBM cell viability was significantly reduced through sh-DLEU1#3 transfection (*P*<0.01), but pcDNA-SP1 (*P*<0.05) or miR-4429 inhibitor (*P*<0.05) countervailed the reduction (Figure 5A). EdU assay proved that DLEU1 deficiency-induced proliferation decline (*P*<0.01) could be reversed by pcDNA-SP1 (*P*<0.05) or miR-4429 inhibitor (*P*<0.05) (Figure 5B). The decrease in GBM cell apoptosis induced by DLEU1 knockdown (*P*<0.01) was abolished through overexpressing SP1 (*P*<0.05) or down-regulating miR-4429 (*P*<0.05) (Figure 5C). In addition, Bax protein level strengthened by silencing DLEU1 was abrogated after enhancing SP1 or lessening miR-4429 expression. The opposite effect was observed in Bcl-2 protein level. Bcl-2 reduction caused by sh-DLEU1#3 could be counteracted via either SP1 overexpression or miR-4429 suppression (Figure 5D). Furthermore, we also detected the impacts of miR-4429 inhibitor or pcDNA-SP1 alone on GBM cell growth. CCK-8 assay suggested that miR-4429 inhibitor or pcDNA-SP1 alone could promote cell viability and decreased the activity of caspase-3 (Supplementary Figure S1A–F). Above results demonstrated that DLEU1 functioned as a positive regulator of GBM growth via miR-4429/SP1.

## Discussion

According to the World Health Organization (WHO) Pathological Grading Standard (2016 version), glioma is admittedly divided into four grades (I–IV) [35]. Mostly, grade I is identified as benign glioma, while diffuse low- and medium-grade gliomas constitute the WHO grade II and III lesions [36]. Grade IV glioma comprises the primary GBM and the secondary glioblastoma from lower grade glioma [37]. GBM, characterized by rapid cell proliferation, is a deadly disease [38,39]. Fortunately, with the development of bioinformatics analysis and medical technology, increasing lncRNAs are detected in GBM and used for the GBM treatment.

Herein, lncRNA DLEU1 is identified to exert the oncogenic function in various cancerous developments. Up-regulation of DLEU1 is closely related to enhanced cell proliferative ability and reduced apoptotic cells in human tumors [13,17]. Currently, we discovered that DLEU1 was up-regulated in GBM tissues from TCGA and in GBM cells. More importantly, we functionally characterized DLEU1 in GBM growth and apoptosis. It turned out that DLEU1 depletion observably decreased GBM cell viability and EdU-positive cells, but strengthened caspase-3 activity and apoptosis-related protein levels. It was hinted that DLEU1 might play an essential role to accelerate GBM development through modulating cell proliferation and apoptosis.

To unravel the mechanism of DLEU1 up-regulation in GBM, we utilized bioinformatics prediction tools (UCSC and JASPAR) since transcription factor has been reported to promote lncRNA’s expression by acting as a transcription activator in cancers [40,41]. We observed that SP1 was a putative transcription factor of DLEU1. Previously, SP1 was reported to transcriptionally induce TMEPAI [42] and lncRNA AGAP2-AS1 [43]. In this work, mechanical experiments disclosed that SP1 bound to the promoter region of DLEU1, thereby activating DLEU1 transcription and up-regulating DLEU1 expression in GBM. Consistently with DLEU1, SP1 was also up-regulated in GBM tissues from TCGA and GBM cells. We speculated that whether DLEU1 could in turn regulate SP1 expression. Previous reports demonstrated that lncRNA could regulate cancerous cellular processes in a ceRNA manner [44,45]. Moreover, we first confirmed that DLEU1 was mainly enriched in cytoplasmic fraction of GBM cells. To dissect the DLEU1-mediated post-transcriptional regulation, we searched the putative miRNAs which could interact with DLEU1. It was found that miR-4429 targeted DLEU1 in GBM cells. MiR-4429 was demonstrated in previous work to act as an anti-tumor miRNA, such as clear cell renal cell carcinoma [46] and papillary thyroid cancer [47]. Our study uncovered that inhibition of miR-4429 aggravated GBM cell growth, consolidating its tumor-suppressive role in GBM. The positive relevance between SP1 and DLEU1 prompted us to further explore the relation of SP1 and miR-4429 in GBM. Applying TargetScan and mechanism experiments, we unveiled that miR-4429 could be sponged by DLEU1 and consequently DLEU1 could elevate SP1 expression through sponging miR-4429. Considering that SP1 facilitated DLEU1 expression, we concluded that SP1/DLEU1/miR-4429 axis constituted a feedback loop in GBM. Shao et al. [48] described that DLEU1 accelerated endometrial cancer development in an SP1-dependent manner via sponging miR-490. Herein, we first corroborated the tumor-facilitating role of DLEU1 depended on SP1 through sponging miR-4429. The interplay between DLEU1 or SP1 and miR-4429 was first presented in GBM. Moreover, we found the SP1 was responsible for DLEUE transcription in GBM. Therefore a positive feedback loop was formed by DLEUE/miR-4429/SP1.

For all we know, our study first proposed that DLEU1 functioned as a ceRNA to release SP1 expression from miR-4429 suppression in GBM. SP1 was a transcription activator of DLEU1, thereby forming a feedback loop. In function, DLEU1 exerted the pro-proliferative and anti-apoptotic potency in GBM cell via miR-4429/SP1 axis. All findings suggested DLEU1 as a promising therapy target for patients with GBM.

## Supplementary Material

Supplementary Figure S1

## Abbreviations

AGAP2-AS1: AGAP2 antisense RNA 1
Ago2: argonaute 2
Bax: Bcl-2 associated X
Bcl-2: mammalian B cell lymphoma-2
CCK-8: cell counting kit-8
cDNA: complementary DNA
ceRNA: competing endogenous RNA
ChIP: chromatin immunoprecipitation
DLEU1: deleted in lymphocytic leukemia 1
DMEM: Dulbecco’s Modified Eagle’s Medium
EdU: 5-ethynyl-20-deoxyuridine
ELK1: ETS transcription factor ELK1
FBS: fetal bovine serum
GAPDH: glyceraldehyde-3-phosphate dehydrogenase
GBM: glioblastoma multiforme
IgG: immunoglobulin G
lncRNA: long non-coding RNA
miRNA: microRNA
OD: optical density
PBS: phosphate-buffered saline
PVDF: polyvinylidene fluoride
qRT-PCR: quantitative real-time polymerase chain reaction
RIP: RNA immunoprecipitation
RIPA: Radio Immunoprecipitation Assay
SDS/PAGE: sodium dodecyl sulfate/polyacrylamide gel electrophoresis
shRNA: short hairpin RNA
SP1: Sp1 transcription factor
TCGA: The Cancer Genome Atlas
TMEPAI: prostate transmembrane protein, androgen induced 1
YY1: YY1 transcription factor
