# Supplementary material for: SP1–DLEU1–miR-4429 feedback loop promotes cell proliferative and anti-apoptotic abilities in human glioblastoma
Source: Biosci Rep. 2019 Dec 6;39(12):BSR20190994. doi: 10.1042/BSR20190994 (PMC6900472; doi:10.1042/BSR20190994)
Supplement: Supplementary Figure S1 [file BSR-2019-0994_supp.pdf]

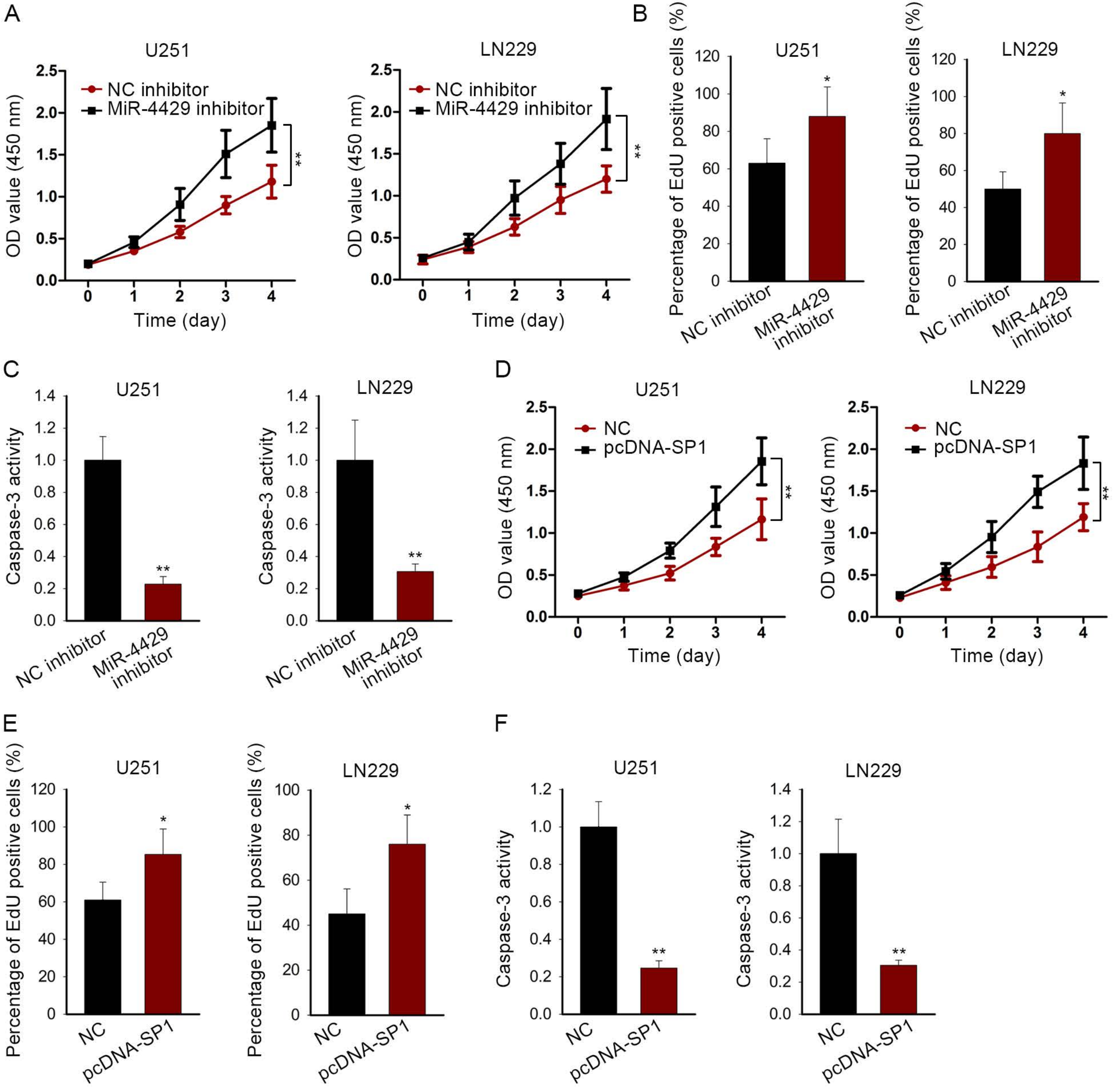

A
U251
LN229
NC inhibitor
MiR-4429 inhibitor
OD value (450 nm)
Time (day)
B
Percentage of EdU positive cells (%)
C
Caspase-3 activity
D
NC
pcDNA-SP1
E
F

Figure S1
A-B. U251 and LN229 cell viability and proliferation were promoted by miR-4429 inhibitor in CCK-8 and EdU assays. **C.** U251 and LN229 cell apoptosis was inhibited by miR-4429 inhibitor in Caspase-3 activity assay. **D-E.** U251 and LN229 cell viability and proliferation were promoted by pcDNA-SP1 in CCK-8 and EdU assays. **F.** U251 and LN229 cell apoptosis was inhibited by pcDNA-SP1 in Caspase-3 activity assay. $p < 0.05$* and $p < 0.01$** are considered to be significant statistically.
